# Is Exposure to Poultry Harmful to Child Nutrition? An Observational Analysis for Rural Ethiopia

**DOI:** 10.1371/journal.pone.0160590

**Published:** 2016-08-16

**Authors:** Derek Headey, Kalle Hirvonen

**Affiliations:** 1 International Food Policy Research Institute, Washington, DC, United States of America; 2 International Food Policy Research Institute, Addis Ababa, Ethiopia; TNO, NETHERLANDS

## Abstract

Although strategic thinking on water, sanitation and hygiene (WASH) has prioritized reducing exposure to human feces in order to limit diarrheal infections, recent research suggests that elevated exposure to livestock–particularly poultry and poultry feces–may be an important risk factor for diarrhea, environmental enteric disorder (EED) and respiratory infections, all of which may seriously retard linear growth in young children. Yet a very different literature on nutrition-sensitive agriculture suggests that livestock ownership is highly beneficial for child growth outcomes through its importance for increasing consumption of nutrient-rich animal sourced foods, such as eggs. Together, these two literatures suggest that the net nutritional benefit of poultry ownership is particularly ambiguous and potentially mediated by whether or not children are highly exposed to poultry. We test this novel hypothesis using a large agricultural survey of rural Ethiopian households that includes measures of child height-for-age Z-scores (HAZ), ownership of poultry and other types of livestock, and an indicator of whether livestock are kept within the main household dwelling overnight. We used least squares regression analysis to estimate unadjusted and adjusted models that control for a wide range of potentially confounding factors. We find that while poultry ownership is positively associated with child HAZ [β = 0.291, s.e. = 0.094], the practice of corralling poultry in the household dwelling overnight is negatively associated with HAZ [β = -0.250, s.e. = 0.118]. Moreover, we find no negative associations between HAZ and corralling other livestock species indoors. These results suggest that while poultry ownership can be beneficial to child growth, overly close exposure to poultry poses a concurrent risk factor for undernutrition, most likely because of increased risk of infection.

## Introduction

Child undernutrition in poor countries has long been linked to disease burdens such as diarrhea and respiratory infections, but more recently to environmental enteric disorder (EED), a sub-clinical condition characterized by chronic damage to the gut, malabsorption of nutrients and low level immune system stimulation that diverts resources away from growth and development [1–3]. Both EED and diarrhea are strongly associated with elevated exposure to fecal matter. But because humans appear to be the main reservoir for several of the most common pathogens that cause clinical diarrhea, the vast majority of water, sanitation and hygiene (WASH) interventions and strategies have placed primary emphasis on toilet construction and related hygiene and water measures, such as handwashing [4–6]. In contrast, there is little indication that WASH or health and nutrition programs regularly include any significant emphasis on reducing exposure to animal feces [7, 8]. Likewise nutrition-sensitive livestock interventions, which are increasingly popular in the developing world because of the important animal-sourced foods for child nutrition [9, 10], pay little or no attention to the health hazards associated with exposure to livestock feces.

There are several reasons to reconsider the neglect of animal feces in the WASH, health/nutrition and agricultural sectors.

First, exposure to animal feces may be more widespread than exposure to human feces in many parts of the developing world. Over 1990–2012, open defecation in the least developed countries fell from 45% to 20%, and in the world as a whole it fell from 24% to 14% [11]. But in most developing countries the majority of rural households own some form of livestock, as do many urban households [12]. While there is no systematic monitoring of the extent to which animal feces pervade homestead environment and private or public water supplies, recent evidence from rural India suggests that animal-sourced fecal matter is much more widespread than human-sourced fecal matter [13].

Second, a recent meta-analysis re-examined linkages between diarrhea in young children and exposure to animals [14]. It found that 21 of 27 suitable studies found significant positive associations between exposure to livestock and diarrhea, even though most of these studies were not specifically looking to corroborate such a linkage. Thus there is some evidence that exposure to livestock does increase the probability of diarrheal infections. Likewise it has long been known that exposure to poultry can increase the risk of respiratory infections in both adults and young children [15].

Finally, it has also been hypothesized that high concentrations of any bacteria–even non-pathogenic bacteria–in the small intestine can cause EED and stunting [1]. So while human feces may contain greater concentrations of the pathogenic bacteria that cause diarrhea [4], the greater prevalence of animal feces in developing countries could potentially pose a greater risk for EED and stunting.

Although biologically plausible, little research has examined the consequences of close exposure to livestock on child nutrition outcomes, even though over half of current and ongoing nutrition-sensitive agricultural projects include some form of livestock intervention [9]. Exceptions include formative research studies from Peru [16], Zimbabwe [17] and Ethiopia [8], in which researchers observed young children directly consuming chicken feces found to contain extremely high concentrations of pathogenic bacteria, such as *Campylobacter jejuni* and *E*. *Coli*. In addition to this formative research, two papers from the Global Enteric Multicenter Study in the Mirzapur sub-district of Bangladesh linked EED and stunting with maternal reports of geophagy [18] and keeping poultry inside the room where young children sleep [19].

In addition to this recent literature on the hygiene dimensions of livestock ownership, there is a re-emerging literature on the important of animal sourced foods (ASFs) for child nutrition in developing countries [20–25], as well as several studies that have attempted to more directly assess the impact of livestock ownership on child nutrition, particularly dairy cattle [26–28]. One recent study also makes a strong case for scaling up egg production and consumption in poor countries on the grounds that eggs are rich in essential fatty acids, proteins, choline, vitamins A and B12, selenium, and other nutrients that are essential for physical and cognitive development [29]. To the best of our knowledge, however, none of this literature has discussed the risks of livestock ownership for gastrointestinal or respiratory infections.

In this study we broach these two separate literatures by exploring the associations between household poultry ownership, exposure of children to poultry in the home, and child height-for-age Z-scores (HAZ) in a large multi-purpose household survey in rural Ethiopia. The context for this observational study, Ethiopia, is apt for several reasons. First, Ethiopia has rates of child stunting in excess of 40 percent [30] and low levels of hygiene, dietary diversity and nutritional knowledge [8, 31–33]. Second, small farms are facing significant financial constraints [34], which inhibits their ability to modernize their livestock systems; hence scavenging systems are still the norm. Third, Ethiopia has one of the highest livestock densities in the world [35], and significant qualitative evidence that Ethiopian children are highly exposed to poultry and their feces [8]. The objective of this study is therefore to explicitly test the hypothesis that poultry ownership in Ethiopia has both a positive association with HAZ (at least in part because poultry ownership increases egg consumption), and a negative association stemming from elevated exposure to livestock-based pathogens.

## Data and Methods

We use household survey data collected by the Central Statistical Agency of Ethiopia, with technical assistance from The International Food Policy Research Institute (for more details of the survey, see [36]). This large scale survey took place between June and July 2015 in the five largest regions of Ethiopia (Amhara, Oromia, Somali, Southern Nations, Nationalities, and Peoples' Region (SNNP) and Tigray). The main purpose of the survey was to obtain post-intervention (midline) information in localities that were to receive investments to improve agricultural production and nutrition under the Feed the Future (FtF) program funded by the United States Agency for International Development (USAID), or in localities that were to act as comparison sites for the evaluation of FtF. These data are representative of the zone of influence within which the FtF program operates but are not regionally or nationally representative. However, the sample is large– 6,977 households–and widespread, the survey having been administered in 252 villages in 84 of Ethiopia’s 670 rural districts (*woredas*).

A novel feature of the survey is that it allows us to link detailed agricultural production with children’s reported intake of food groups (dietary diversity) in the previous 24 hours and anthropometry. Anthropometric and dietary diversity data were collected for preschool children 0–59 months of age, amounting to a final sample of 3,494 children (from 2,704 households) after data cleaning. Length or height-for-age Z-scores (hereafter HAZ)–measured against World Health Organization norms [37]–constitute our main nutrition outcome of interest. Mothers were also asked whether children consumed particular foods over the last 24 hours, including a range of animal-sourced foods (ASFs) categorized into broader groups such as dairy products, eggs and flesh foods. The survey also records a number of other indicators typically present in nutrition models, including a standardized household asset index and various separate housing characteristics (electricity, roof materials), ownership of agricultural equipment, farm sizes, the highest level of parental education, access to an improved water source, household toilet use, access to health and agricultural extension services, and a series of questions pertaining to nutritional knowledge of appropriate child feeding practices from which we construct a nutritional knowledge z-score using principal components analysis.

In terms of livestock the FtF survey records ownership of different types of livestock types in addition to poultry. However, in earlier field visits we observed that while some household kept poultry in separate chicken coops/houses at night, many households keep poultry (and other livestock) in their main dwelling overnight, which may imply elevated levels of exposure to pathogens. Hence we requested the inclusion of additional questions to the FtF midline survey on whether any animals were kept inside the housing structure where household members sleep. This question results in indicators similar to those used in the Global Enteric Multicenter Study in Bangladesh [19], except that in the FtF, the question was posed for all types of livestock (as opposed to poultry only). A limitation of this question is that it may underestimate children’s exposure to poultry and poultry feces given that scavenging poultry rarely venture further than 50 meters away from the homestead [38], and may therefore contaminate outdoor areas where children sit or crawl. However, we hypothesized that keeping poultry in the main household dwelling overnight might offer additional contamination in indoor areas where children spend considerable amount of time (especially in the rainy season). Hence we test whether the practice of keeping poultry and other livestock in the household itself poses a specific risk for children, whilst acknowledging that there are still risks even for households that keep scavenging poultry in external chicken houses.

**Fig 1:**
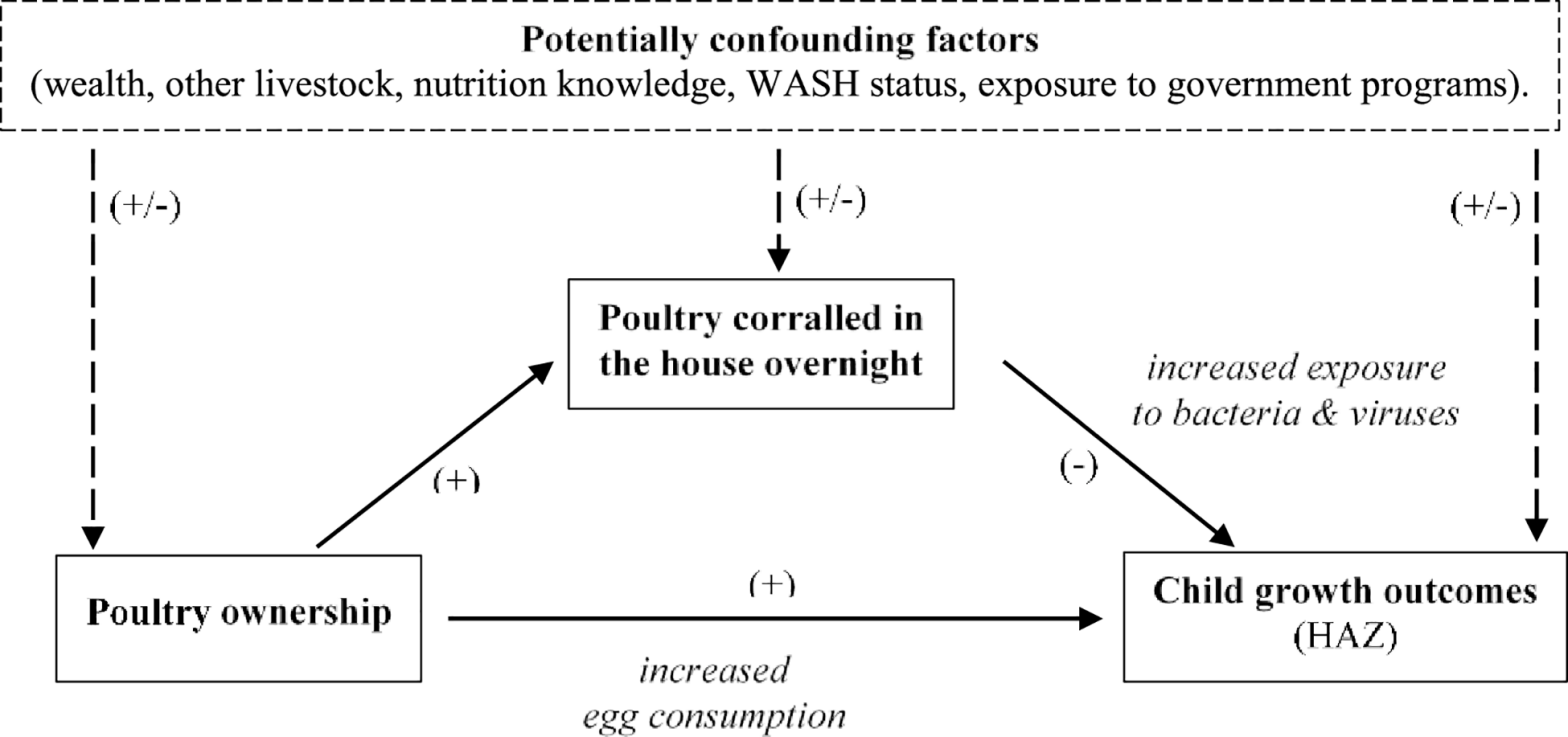
A conceptual framework linking poultry ownership to child growth, with poultry corralling indoors as a mediating factor. Source: Authors’ construction.

Fig 1 describes the principal hypothesis underlying the analytical framework of this study. As noted in the introduction, livestock ownership in remote rural areas is generally hypothesized to be an important precondition for increasing ASF consumption, which in the case of poultry would primarily refer to egg consumption. However, we additionally hypothesize that the positive relationship between poultry ownership and child growth is negatively mediated by children’s physical exposure to poultry (operationalized by whether livestock are corralled in the house overnight), which increases the risk of contact with harmful pathogens. A simple means of testing this mediation hypothesis is to first estimate a least squares regression model of HAZ as a function of poultry ownership alone, and then to add the poultry exposure indicator to a second model [39]. If the coefficient on poultry being kept indoors is significant and negative, and the coefficient on poultry ownership is reduced in magnitude in the second model, this would be consistent with elevated exposure to poultry being a mediating factor in the livestock-HAZ relationship.

At the same time, the dashed box and dashed arrows in Fig 1 illustrate the risk of confounding factors simultaneously influencing the decision to own poultry, the decision to corral poultry indoors, and child growth outcomes. In particular, the decisions to own poultry or corral them indoors could be related to household wealth (household assets, agricultural assets such as farm size or ownership of other livestock), parental education and nutritional knowledge, general household hygiene (toilet use, improved water supply), housing characteristics (floor and roof materials), household demographics, or environmental factors (such as overnight temperatures or rainfall, or exposure to animal predators or theft). Fortunately the multipurpose nature of our survey–and the use of village fixed effects to capture time-invariant environmental factors–allows us to control for these kinds of confounding factors (referred to as "adjusted models" below), and to test whether they systematically vary across households that do or do not own poultry, and do or do not corral their poultry indoors. Thus while the data and study design at hand is not experimental by design, we can at least test the robustness of the hypothesized model in Fig 1 to the presence of potentially confounding factors.

Finally, while the objective of our empirical analysis is to test for negative mediation of children’s exposure to poultry, the FtF data set can also be used to engage in supplementary tests of whether poultry ownership increases children’s egg consumption. To test this we follow a similar analysis of cattle ownership, dairy consumption and child nutrition in rural Ethiopia [27]. First, we use a linear probability model to estimate egg consumption as a function of poultry ownership after controlling for the confounding factors referred to in Fig 1. Second, we test whether poultry ownership might also influence children’s diets through generic wealth effects, rather than specifically through egg availability. To do so we regress indicators of children’s consumption of non-egg ASFs against poultry ownership and control variables. Finally, we explore how closely indicators of socioeconomic status (household assets, farm size, electricity access, housing materials and non-poultry ASF consumption) are related to poultry ownership after controlling for other factors. These three supplementary tests can therefore shed light on whether the positive associations between HAZ and poultry ownership stem from direct effects on egg consumption and/or more generic linkages between household wealth and poultry ownership (linkages which could be bi-directional).

## Descriptive Results

We begin our analysis with some descriptive analysis of nutrition, diets and animal husbandry in rural Ethiopia. Livestock ownership and housing practices are reported in Table 1. As expected, we find that ownership rates of livestock are very high (with relatively little regional variation; see S1 Table in our supplement). Consistent with the nationally representative 2011 Ethiopian Demographic Health Survey [30], the FtF midline survey suggests that around half of rural households own poultry. We also observe that keeping livestock indoors at night is very common, particularly for poultry owners, almost half of whom follow this practice (48%).

**Table 1:** Livestock ownership and corralling practices by livestock type in a sample of 2,704 rural Ethiopian households.

Livestock type	Livestock ownership (% of households)	Among livestock owners, the percentage who corralled animals in the main house overnight ^a^
Poultry	48%	48%
Bulls, oxen	58%	23%
Cows	63%	26%
Calves, heifers	66%	36%
Goats, sheep	52%	31%
Pack animals	42%	18%

Notes

^a.^ This question specifically asks whether each type of livestock is typically kept overnight in the structure where household members sleep.

Table 2 reports means of the main variables used in our analysis for the sample as a whole as well as sub-samples based on whether the household owns no poultry, owns poultry that are corralled inside the main household dwelling overnight, or owns poultry that are corralled outside the main dwelling. We also report t-tests for differences in means relative to the sub-sample of households that keep poultry inside the main household dwelling ("Poultry inside"). These t-tests provide some indicative evidence as to whether confounding factors may influence the associations hypothesized in Fig 1.

**Table 2:** Variable means for the full sample and poultry-based sub-samples, including t-tests of mean differences relative to the "Poultry inside" sub-sample.

	All households	No poultry	Poultry outside	Poultry inside
	(N = 3,934 children 0–59 months of age from 2,704 households)	(N = 1,835 children 0–59 months of age from 1,415 households)	(N = 860 children 0–59 months of age from 671 households)	(N = 799 children 0–59 months of age from 618 households)
HAZ score	-1.75	-1.82	-1.56***	-1.80
Stunting (%)	48.43	50.08	43.49***	49.94
ASF in last 24 hours (%)	31.25	30.82	31.28	32.23
Eggs in last 24 hours (%)	4.58	2.07***	8.95***	5.64
Number of poultry owned	1.96	n/a	4.39***	3.82
Tropical livestock units (TLUs)	3.60	3.41	4.06***	3.57
**Control variables in regression models:**				
Owns other livestock (%)	90.67	87.47***	94.65	93.74
Other livestock kept inside (%)	32.86	30.95***	9.65***	62.20
Highest education (years)	4.06	3.66***	4.87	4.12
Household assets (birr), log	6.88	6.55***	7.42	7.03
Land size (acres), log	0.65	0.50***	0.92	0.69
Iron roof (%)	42.59	37.11***	51.16	45.93
Dirt, mud or dung floor (%)	97.02	96.73***	96.16***	98.62
Uses toilet (%)	61.22	57.49***	63.26	67.58
Safe water (%)	59.59	57.06**	63.37	61.33
Electricity (%)	6.41	7.08***	6.86**	4.38
Nutrition knowledge z score	0.00	-0.09***	0.13	0.07
Health worker visited (%)	22.84	19.13***	25.12*	28.91
Agricultural worker visited (%)	29.48	25.94***	30.58**	36.42
Child age (months)	33.48	33.56	33.35	33.44
Female (%)	50.57	48.50**	52.91	52.82
Muslim (%)	28.33	37.28***	17.67	19.27
Orthodox Christian (%)	44.13	34.71***	61.16***	47.43
Other religion (%)	27.53	28.01***	21.16***	33.29

Notes

*, ** and *** indicate significance at the 10%, 5% and 1% level, respectively, for tests of whether "no poultry" and "poultry outside" have common means to the "poultry inside" sample. This is a two-tailed t-test based on unequal variances.

In terms of anthropometric outcomes, Table 1 shows that mean HAZ scores are very low, and almost half (48%) of our sample of children are stunted (HAZ below two standard deviations), a result consistent with results of recent Demographic Health Surveys in Ethiopia [40]. Furthermore, S1 Fig in our supplement shows the HAZ scores in this sample follow the usual dynamics of growth faltering observed in other low income country settings [41].

In Table 2 we also observe that the sub-sample of "poultry outside" has significantly better nutritional outcomes (44% stunting) compared to "poultry inside" (50% stunting). ASF consumption is low and not significantly different across groups, but egg consumption by children is higher in the poultry-owning groups, and higher in the "poultry outside" sub-sample than in the "poultry inside" group. However, we subsequently show below that this difference in egg consumption between the "poultry outside" and "poultry inside" groups is not significant after other factors are taken into account.

We also observe another significant difference across the sub-samples: "Poultry inside" households are more likely to keep other animals inside and own more livestock (perhaps because of common threats like predation or theft). These relationships are further explored in S2 Table in our supplement. However, between "poultry inside" and "poultry outside" households there are no significant differences in non-livestock indicators like education, assets, land size, toilet use, safe water, nutritional knowledge, or child age and sex, even though "poultry outside" households are slightly less likely to receive health or agricultural extension visits. Interestingly, although poultry is regarded as a low-entry activity that even the poor can engage in [42], we generally observe that households not owning poultry are somewhat poorer and less educated than those owning poultry. Lastly, we also know that there are some spatial differences between the "poultry inside" and "poultry outside" samples, including altitude, which strongly influences night-time temperature (results available on request). However, controlling for village fixed effects eliminates these time-invariant confounding factors from spuriously influencing the main relationships of interest. Moreover, the intra-cluster correlation of "poultry inside" is quite low (0.22), suggesting that there is plenty of within-village variation in whether households keep poultry indoors.

## Regression Results

Table 3 reports our HAZ regression results for 3,494 children aged 0–59 months. Regression 1 specifies an unadjusted model that only includes poultry ownership, along with only very basic controls for child age (linear spline function [43] with knots at 12 and 24 months) and sex, as well as village fixed effects. Regression 2 estimates the same unadjusted model with the addition of the binary variable for whether a household keeps poultry in the main house overnight. Comparison of "Owns poultry" coefficients across these two models provides a test of whether "Poultry in house" is a mediating factor in the relationship between "Owns poultry" and child HAZ. In regression 1 we observe a highly significant and positive coefficient on "Owns poultry" equal to 0.207. In regression 2 this coefficient increases to 0.346 and remains highly significant, while the addition of "Poultry in house" to the model in Regression 2 also yields a statistically significant coefficient, but one that is negative and large in magnitude (-0.286). Regression 2 therefore suggests that keeping poultry in close proximity to children is an important negative mediator that substantially reduces the positive association between poultry ownership and HAZ. Indeed, in regression 2 we cannot reject the null hypothesis that the coefficients on "Owns poultry" and "Poultry in house" are equal in absolute magnitude (p = 0.531), suggesting that keeping poultry in close proximity to children could substantially offset the growth benefits of household poultry ownership.

**Table 3:** Adjusted and unadjusted least squares regression models of child HAZ scores against binary indicators of "Owns poultry" and "Poultry in house".

	(1)	(2)	(3)	(4)
	Unadjusted	Unadjusted	Adjusted	Adjusted
	N = 3,494	N = 3,494	N = 3,494	N = 3,494
Owns poultry (0/1)	0.207***	0.346***	0.168**	0.291***
	(0.074)	(0.093)	(0.074)	(0.094)
Poultry in house (0/1)		-0.286**		-0.250**
		(0.116)		(0.118)
Owns other livestock (0/1)			0.171	0.148
			(0.109)	(0.109)
Other livestock in house (0/1)			0.015	0.070
			(0.090)	(0.092)
Highest education (years)			0.034***	0.033***
			(0.012)	(0.012)
Household assets (birr), log			0.038	0.037
			(0.032)	(0.032)
Land size (acres), log			-0.055	-0.055
			(0.044)	(0.044)
Iron roof (0/1)			0.175*	0.164
			(0.100)	(0.100)
Uses toilet (0/1)			0.094	0.090
			(0.083)	(0.084)
Safe water (0/1)			0.020	0.014
			(0.104)	(0.104)
Electricity (0/1)			-0.041	-0.048
			(0.193)	(0.193)
Earth, mud or dung floor (0/1)			-0.075	-0.074
			(0.207)	(0.206)
Nutrition knowledge z score			0.019	0.018
			(0.041)	(0.041)
Health worker visited (0/1)			0.077	0.080
			(0.078)	(0.078)
Agricultural worker visited (0/1)			0.149*	0.147*
			(0.079)	(0.079)
Child age and sex controls?	Yes	Yes	Yes	Yes
Village fixed effects?	Yes	Yes	Yes	Yes
Household demographic controls?	No	No	Yes	Yes
R-squared	0.136	0.138	0.149	0.151

Notes: Standard errors clustered at the village level are reported in parentheses.

*, ** and *** indicate significance at the 10%, 5% and 1% level, respectively. See Section 2 for descriptions of the variables.

In regressions 3 and 4 we repeat the same exercise but adjust for the potential confounding factors listed in Table 2. The effect of introducing these controls is to mildly reduce the magnitudes of the coefficients on "Owns poultry" and "Poultry in house", but the inferences above remain unchanged. In the adjusted model in regression 4 for example, the coefficients on "Owns poultry" and "Poultry in house" are both large in absolute magnitude, but an F-test confirms that the two coefficients are not significantly different from each other in absolute terms (p = 0.669). Observable confounding factors therefore do not seem to substantially influence the associations hypothesized in Fig 1.

One other testable implication of the associations hypothesized in Fig 1 is the assumption that poultry ownership is beneficial to child nutrition through increased consumption of eggs, which are nutritionally very beneficial for young children [29], rather than simply being an indicator of generic household wealth. In regression 1 of Table 4 we therefore estimate a linear probability model with 24-hour recall of egg consumption as the dependent variable. As hypothesized, poultry ownership increases the probability of egg consumption, with the point estimate suggesting an effect of 5.5 percentage points. Also of note is that the coefficient on "Poultry inside" is insignificant, thus assuaging a concern raised by the t-tests of sub-sample means in Table 2, that children from households who keep poultry outside are somewhat more likely to have consumed egg in the past 24 hours.

**Table 4:** Linear probability models for binary indicators of 24-hour recall of children’s ASF consumption.

	1	2	3	4
	N = 3,493	N = 3,446	N = 3,466	N = 3,473
Dependent variable	consumed eggs	consumed non-egg ASF	consumed any meat	consumed dairy
Owns poultry (0/1)	0.055***	0.018	0.005	0.016
	(0.014)	(0.022)	(0.012)	(0.023)
Poultry in house (0/1)	-0.016	-0.004	-0.013	0.001
	(0.018)	(0.031)	(0.012)	(0.031)
Owns other livestock (0/1)	0.013	0.096***	0.007	0.088***
	(0.015)	(0.031)	(0.018)	(0.030)
Other livestock in house (0/1)	0.006	0.039	0.000	0.028
	(0.011)	(0.026)	(0.011)	(0.025)
Highest education (years)	0.004***	0.001	-0.002	0.003
	(0.002)	(0.003)	(0.002)	(0.003)
Household assets (birr), log	0.008**	0.030***	0.011***	0.023***
	(0.004)	(0.007)	(0.003)	(0.007)
Land size (acres), log	0.008	0.030***	0.012**	0.028***
	(0.005)	(0.011)	(0.006)	(0.011)
Iron roof (0/1)	0.007	0.009	-0.010	0.009
	(0.013)	(0.023)	(0.010)	(0.023)
Uses toilet (0/1)	0.015*	-0.027	0.008	-0.037
	(0.009)	(0.026)	(0.010)	(0.024)
Safe water (0/1)	-0.010	0.027	0.002	0.020
	(0.010)	(0.023)	(0.011)	(0.023)
Electricity (0/1)	0.042*	0.145***	0.055**	0.121**
	(0.024)	(0.047)	(0.026)	(0.049)
Earth, mud or dung floor (0/1)	0.008	0.014	-0.033	0.027
	(0.018)	(0.048)	(0.027)	(0.046)
Nutrition knowledge z score	-0.003	0.010	0.010*	0.007
	(0.005)	(0.009)	(0.005)	(0.009)
Health worker visited (0/1)	0.023**	0.026	0.012	0.027
	(0.011)	(0.022)	(0.013)	(0.021)
Agricultural worker visited (0/1)	0.008	0.008	-0.001	0.004
	(0.011)	(0.023)	(0.011)	(0.023)
R-squared	0.180	0.252	0.207	0.257

Notes: These are linear probability regressions. Standard errors are reported in parentheses, and are clustered at the village level.

*, ** and *** indicate significance at the 10%, 5% and 1% level, respectively. See Section 2 for descriptions of the variables. All regressions include controls for child and household demographics and religion, as well as village fixed effects.

The remaining regressions in Table 4 explore whether poultry ownership might influence HAZ through consumption of non-egg ASFs, particularly if poultry ownership is a strong indicator of rural wealth. However, we find no evidence that poultry ownership or keeping poultry inside the house are significantly associated with the consumption of non-eggs ASFs such as meat or dairy products. In supplemental results, however, we do find that poultry ownership is indeed associated with indicators of household socioeconomic status (S3 Table in our supplement). The direction of causation in this relationship is ambiguous: household wealth could increase the likelihood of owning poultry, or poultry ownership could contribute to household wealth accumulation. Nevertheless, it is comforting that the coefficient on the "Poultry in the house" variable remains insignificant in all models implying that there are no statistically significant wealth differences between "Poultry-in" and Poultry-out" households, after controlling for other factors. Finally, the fact that poultry ownership does increase the probability of children’s consumption of eggs (Table 4) suggests that the association between poultry ownership and HAZ is at least plausibly causal.

In addition to the results in Tables 3 and 4 we engaged in a number of other robustness tests, which are reported in the Supplementary Materials. For example, one concern with the results in Table 3 is that all types of non-poultry livestock are aggregated together. One hypothesis might be that that small ruminants (sheep, goats) also contaminate areas where young children spend much of their time, whereas larger ruminants spend more time further from the house. To explore this, in Table 5 we disaggregate "other livestock" into small ruminants (sheep and goats) and larger animals (cattle and pack animals), but we do not uncover significant coefficients for these categories, and the poultry coefficients remain materially unchanged.

**Table 5:** Adjusted least squares regression models of child HAZ scores against binary indicators of "Owns poultry" and "Poultry in house" and a series of more disaggregated livestock categories.

	(1)	(2)
	N = 3,494	N = 3,494
Owns poultry (0/1)	0.291***	0.301***
	(0.094)	(0.095)
Poultry kept in house (0/1)	-0.250**	-0.261**
	(0.118)	(0.118)
Owns other livestock (0/1)	0.148	
	(0.109)	
Other livestock kept in house (0/1)	0.065	
	(0.091)	
Owns sheep/goats (0/1)		-0.032
		(0.074)
Sheep/goats kept in house (0/1)		0.082
		(0.125)
Owns cattle/pack animals (0/1)		0.112
		(0.102)
Keeps cattle/pack animals in house (0/1)		0.060
		(0.101)
All other controls included?	Yes	Yes
R-squared	0.151	0.151

Notes: Standard errors are reported in parentheses, and are clustered at the village level.

*, ** and *** indicate significance at the 10%, 5% and 1% level, respectively. Control variables included in the model but omitted from the table include demographic controls, village fixed effects, child sex and child age.

We also tested the robustness of the results in Table 3 to stepwise addition of control variables (S4 Table in our supplement). The coefficients on the "Owns Poultry" and "Poultry in house" variables remain remarkably stable across these different specifications.

Lastly, we explored whether keeping livestock in the house had varying associations at different points in the HAZ distribution. Least squares regressions minimize variation around the mean, which is very low in Ethiopia, but researchers often also use dichotomous variables as dependent variables such HAZ<-2. Though intuitive in the terms of quantifying odds ratios for risk factors, using dichotomous variables unnecessarily discards information and is not recommended by epidemiological statisticians [44]. We therefore used a series of quantile regressions (for previous examples, see [45] and [27] in the context of nutrition) that minimize absolute residual deviations around the 25^th^, 50^th^, 75^th^ quantiles to explore whether keeping livestock in the house had different impacts at different points in the conditional HAZ distribution (S5 Table in our supplement). We find some tendency towards stronger relationships when the regressions minimize deviations around higher levels of HAZ. However, comparison of the point estimates between the 25^th^ and 75^th^ quantiles reveals that the differences are not statistically significant. Hence irrespective of the quantile in question, the regressions continue to suggest that keeping poultry inside the house has large adverse consequences for child growth.

## Discussion

This paper engaged in an observational analysis of the novel hypothesis that the otherwise positive relationship between poultry ownership and child nutrition is negatively mediated by a child’s physical proximity to poultry, as operationalized by whether a household keeps poultry indoors overnight. The results reported in the previous section are consistent with this hypothesis. Although these associations should not be interpreted too literally, the negative association between close physical exposure to poultry and child growth is large in magnitude, and not statistically different in magnitude from the positive association between poultry ownership and growth. This results suggests that poultry-related hygiene issues are an important mediating factor linking poultry ownership to child growth.

Inferences from these results should be cautious, because the data and methods used to reach these conclusions come with several important caveats. First, the coefficient estimates reported above are associations derived from an exploratory analysis of observational data rather than causal estimates from a confirmatory analysis. That said, we found no evidence that the decision of poultry-owning households to keep livestock indoors or outdoors is associated with any observable factors, nor were the adjusted regression models in Table 3 unduly sensitive to the inclusion of potential confounding variables. Second, although the presence of poultry in the house appears to be a relevant indicator of proximity of poultry, as also indicated by the Bangladesh study cited above [19], this is an only an indirect indicator of exposure to animal fecal matter or other livestock-related disease vectors. Indeed, measurement error in this respect might cause a downwards attenuation bias in our estimates; that is, more accurate measures of exposure to animal feces might reveal stronger negative associations. Third, while child growth is in many respects a reasonable biomarker for capturing long term exposure to infections, the large scale FtF survey used in this study does not include biomarkers of environmental enteropathy, appropriate indicators of diarrheal or chest infections, alternative nutrition outcomes of potential relevance, or indicators of cognitive performance.

These limitations aside, this study makes two important contributions to the existing literature on child nutrition, as well as the various literatures on WASH, health/nutrition and livestock interventions in developing countries.

First, the results corroborate findings from the more qualitative research studies cited above in which small samples of young children were observed over long periods [8, 16, 17, 19]. These studies observed that young children in developing countries are highly exposed to poultry feces and the animals themselves. We also find that large proportions of young children are highly exposed to poultry in terms of sheer physical proximity. One of these studies also hypothesized that this exposure might lead to adverse nutrition outcomes through EED [17], but was not able to demonstrate an adverse impact on HAZ due to sampling limitations; our study shows that this exposure at least predicts reductions in child growth, even if the biological mechanisms for such an association cannot be identified with the data at hand. Our principal finding is similar to the MAL-ED studies from Bangladesh, which found that keeping poultry indoors and maternal reports of child geophagy were both associated with child stunting, as well as EED markers [18, 19]. Two differences of note are: (1) the MAL-ED studies only collected data for a small sample of poultry-owning households (N = 217); and (2) these studies did not explore whether poultry ownership also had positive associations with stunting after controlling for poultry exposure or geophagy.

Second, the studies cited above all suggest that poultry appear to pose a special risk to children. Although it is certainly conceivable that exposure to other livestock yields similar risks, existing studies yield plausible indications as to why poultry might pose a much greater risk. In Ethiopia, as in most poor settings, poultry are scavenging because of the relative expense of purchasing improved feed (feed being an essential precondition for corralling livestock). In scavenging poultry systems the flock tends to roam in and around the household dwelling (within 50 meters) during the day in pursuit of household food waste [38]. As a result, poultry tend to defecate in and around the homestead where young children are also left to sit or crawl, while other livestock likely defecate much further from the homestead. Moreover, poultry feces are small enough to be easily handled and ingested by small children, and small, dry and odorless enough to be neglected by caregivers (unlike larger animals). And as noted in the introduction, poultry feces have high concentrations of pathogenic bacteria [16, 17] and poultry have long been identified as potentially important vectors for respiratory infections [15]. Hence, there are several behavioral and biological reasons to believe that poultry ownership poses a particularly acute health risk for young children.

Finally, the results reported in Section 4 have important implications for programs and policies across several sectors. While it has been argued that egg consumption has significant potential to improve child nutrition outcomes in developing countries [29], the results of this study suggest that poultry interventions in developing countries could yield much greater health benefits for young children if they focused greater attention on improving household hygiene knowledge, and on improving livestock management practices, particularly the reduction of children’s physical exposure to poultry and their feces [4, 46, 47]. The same message applies to WASH strategies, which have historically placed primary emphasis on reducing exposure to human rather than animal feces [4]. Finally, the potential for nutritional synergies between livestock management, household hygiene, and infant and young child care practices, suggests the need for greater coordination between several sectors–WASH, health/nutrition and agriculture–that have often interacted very little with each other. The results in this paper therefore provide further justification for emphasizing the importance of multisectoral coordination in combating child undernutrition [48].

## Supporting Information

S1 Fig Height-for-age Z-score by child age among a sample of 3,494 children. (DOCX)

S1 Table Mean livestock ownership by livestock type and region for 2,704 rural Ethiopian households. (DOCX)

S2 Table Correlations between binary indicators of which animals are kept in the main household dwelling overnight among 2,704 rural Ethiopian households. (DOCX)

S3 Table Least squares regression models of socioeconomic indicators as a function of poultry indicators and control variables. (DOCX)

S4 Table Testing the robustness of the results in Table 3 to stepwise addition of control variables. (DOCX)

S5 Table Adjusted quantile regression models of child HAZ scores against binary indicators of "Owns poultry" and "Poultry in house". (DOCX)
