# Supplementary material for: Is Exposure to Poultry Harmful to Child Nutrition? An Observational Analysis for Rural Ethiopia
Source: PLoS One. 2016 Aug 16;11(8):e0160590. doi: 10.1371/journal.pone.0160590 (PMC4986937; doi:10.1371/journal.pone.0160590)
Supplement: S1 Fig — (DOCX) [file pone.0160590.s001.docx]

**S1 Fig: Height-for-age Z-score by child age among a sample of 3,494 children**


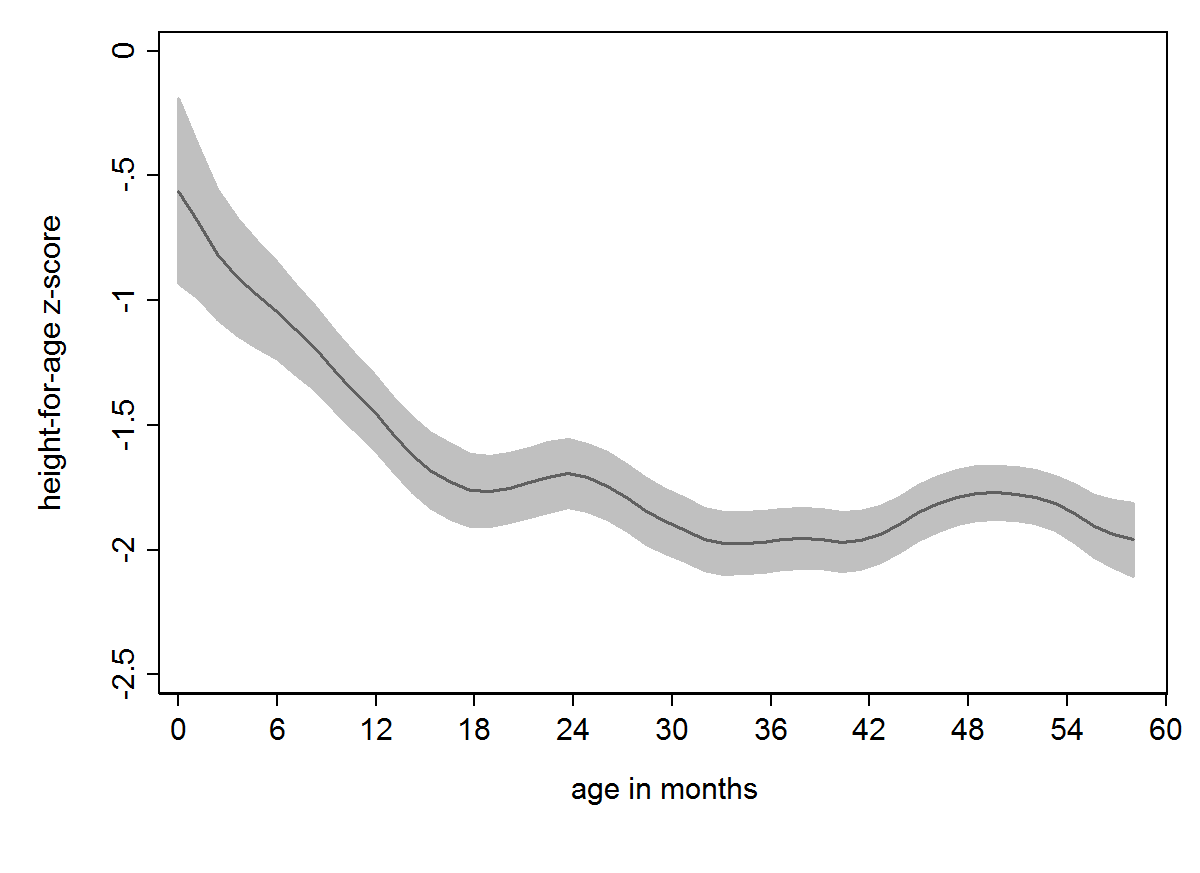


Notes: This predicted relationship is generated by a local polynomial kernel smoother

with 95% confidence intervals.
