# Supplementary material for: Is Exposure to Poultry Harmful to Child Nutrition? An Observational Analysis for Rural Ethiopia
Source: PLoS One. 2016 Aug 16;11(8):e0160590. doi: 10.1371/journal.pone.0160590 (PMC4986937; doi:10.1371/journal.pone.0160590)
Supplement: S1 Table — (DOCX) [file pone.0160590.s002.docx]

**S1 Table. Mean livestock ownership by livestock type and region for 2,704 rural Ethiopian households**

| **Livestock type** | **All areas** | **Tigray** | **Amhara** | **Oromia** | **Somale** | **SNNP** |
| --- | --- | --- | --- | --- | --- | --- |
| Poultry | 48% | 67% | 55% | 48% | 14% | 41% |
| Bulls, oxen | 58% | 76% | 76% | 58% | 21% | 41% |
| Cows | 63% | 65% | 61% | 64% | 56% | 65% |
| Calves, heifers | 66% | 72% | 51% | 53% | 72% | 51% |
| Goats, sheep | 52% | 69% | 48% | 46% | 69% | 48% |
| Pack animals | 42% | 67% | 53% | 46% | 67% | 53% |
