# Supplementary material for: Is Exposure to Poultry Harmful to Child Nutrition? An Observational Analysis for Rural Ethiopia
Source: PLoS One. 2016 Aug 16;11(8):e0160590. doi: 10.1371/journal.pone.0160590 (PMC4986937; doi:10.1371/journal.pone.0160590)
Supplement: S2 Table — (DOCX) [file pone.0160590.s003.docx]

**S2 Table: Correlations between binary indicators of which animals are kept in the main household dwelling overnight among 2,704 rural Ethiopian households**

|  | calves inside | bulls inside | oxen inside | cows inside | sheep/goats inside | pack animals inside |
| --- | --- | --- | --- | --- | --- | --- |
| bulls inside | 0.175* |  |  |  |  |  |
| oxen inside | 0.468* | 0.096* |  |  |  |  |
| cows inside | 0.623* | 0.141* | 0.540* |  |  |  |
| sheep/goats inside | 0.399* | 0.059* | 0.312* | 0.384* |  |  |
| pack animals inside | 0.339* | 0.058* | 0.414* | 0.367* | 0.332* |  |
| chicken inside | 0.251* | 0.075* | 0.188* | 0.181* | 0.201* | 0.180* |

Notes: * indicates significant at the 1% level
