# Supplementary material for: Is Exposure to Poultry Harmful to Child Nutrition? An Observational Analysis for Rural Ethiopia
Source: PLoS One. 2016 Aug 16;11(8):e0160590. doi: 10.1371/journal.pone.0160590 (PMC4986937; doi:10.1371/journal.pone.0160590)
Supplement: S3 Table — (DOCX) [file pone.0160590.s004.docx]

**S3 Table. Least squares regression models of socioeconomic indicators as a function of poultry indicators and control variables**

|  | **log of household durables (birr)** | **log of farm size (hectares)** | **Electricity (0/1)** |
| --- | --- | --- | --- |
|  | N=3,494 | N=3,494 | N=3,494 |
|  |  |  |  |
| Owns poultry | 0.285*** | 0.123** | -0.005 |
|  | (0.074) | (0.051) | (0.011) |
| Poultry in house | -0.128 | -0.025 | -0.016 |
|  | (0.081) | (0.057) | (0.013) |
| Owns other livestock | 0.560*** | 0.420*** | 0.011 |
|  | (0.128) | (0.091) | (0.018) |
| Other livestock in house | -0.276*** | -0.062 | -0.026** |
|  | (0.072) | (0.048) | (0.011) |
| Highest education (years) | 0.082*** | -0.001 | 0.006*** |
|  | (0.011) | (0.007) | (0.002) |
| Nutrition knowledge z score | 0.079** | -0.004 | 0.009* |
|  | (0.033) | (0.023) | (0.005) |
| Health worker/volunteer visited | 0.011 | -0.003 | 0.001 |
|  | (0.066) | (0.056) | (0.010) |
| Agricultural worker visited | 0.036 | 0.176*** | 0.011 |
|  | (0.059) | (0.042) | (0.011) |
|  |  |  |  |
| Child age and sex controls? | Yes | Yes | Yes |
| Village fixed effects? | Yes | Yes | Yes |
| Household demographic controls? | Yes | Yes | Yes |
| R-squared | 0.570 | 0.545 | 0.487 |

Notes: Standard errors are reported in parentheses, and are clustered at the village level. *, ** and *** indicate significance at the 10%, 5% and 1% level, respectively. See Section 2 for descriptions of the variables. All columns include controls for child age and sex, village fixed effects and demographic controls.
