# Supplementary material for: Is Exposure to Poultry Harmful to Child Nutrition? An Observational Analysis for Rural Ethiopia
Source: PLoS One. 2016 Aug 16;11(8):e0160590. doi: 10.1371/journal.pone.0160590 (PMC4986937; doi:10.1371/journal.pone.0160590)
Supplement: S4 Table — (DOCX) [file pone.0160590.s005.docx]

**S4 Table: Testing the robustness of the results in Table 3 to stepwise addition of control variables**

|  | **(1)** | **(2)** | **(3)** | **(4)** | **(5)** | **(6)** |
| --- | --- | --- | --- | --- | --- | --- |
|  | N=3,494 | N=3,494 | N=3,494 | N=3,494 | N=3,494 | N=3,494 |
|  |  |  |  |  |  |  |
| Owns poultry (0/1) | 0.244*** | 0.243*** | 0.328*** | 0.318*** | 0.298*** | 0.291*** |
|  | (0.086) | (0.088) | (0.096) | (0.093) | (0.092) | (0.092) |
| Poultry in house (0/1) | -0.239** | -0.251** | -0.284** | -0.262** | -0.232** | -0.230** |
|  | (0.105) | (0.109) | (0.118) | (0.117) | (0.115) | (0.115) |
| Owns other livestock (0/1) |  | 0.09 | 0.214** |  |  |  |
|  |  | (0.103) | (0.105) |  |  |  |
| Other livestock in house (0/1) |  | 0.025 | 0.007 |  |  |  |
|  |  | (0.081) | (0.091) |  |  |  |
| Highest education (years) |  |  |  | 0.040*** | 0.033*** | 0.033*** |
|  |  |  |  | (0.011) | (0.012) | (0.012) |
| Household assets (birr), log |  |  |  |  | 0.04 | 0.039 |
|  |  |  |  |  | (0.031) | (0.031) |
| Land size (acres), log |  |  |  |  | -0.042 | -0.048 |
|  |  |  |  |  | (0.044) | (0.044) |
| Iron roof (0/1) |  |  |  |  | 0.16 | 0.157 |
|  |  |  |  |  | (0.099) | (0.099) |
| Uses toilet (0/1) |  |  |  |  | 0.1 | 0.087 |
|  |  |  |  |  | (0.084) | (0.084) |
| Safe water (0/1) |  |  |  |  | 0.019 | 0.013 |
|  |  |  |  |  | (0.104) | (0.104) |
| Electricity (0/1) |  |  |  |  | -0.042 | -0.052 |
|  |  |  |  |  | (0.187) | (0.192) |
| Earth, mud or dung floor (0/1) |  |  |  |  | -0.066 | -0.06 |
|  |  |  |  |  | (0.207) | (0.207) |
| Nutrition knowledge z score |  |  |  |  |  | 0.016 |
|  |  |  |  |  |  | (0.041) |
| Health worker visited (0/1) |  |  |  |  |  | 0.077 |
|  |  |  |  |  |  | (0.079) |
| Agricultural worker visited (0/1) |  |  |  |  |  | 0.152* |
|  |  |  |  |  |  | (0.079) |
| Child age and sex controls? | Yes | Yes | Yes | Yes | Yes | Yes |
| Village fixed effects? | No | No | Yes | Yes | Yes | Yes |
| Household demographic controls? | No | No | No | Yes | Yes | Yes |
| R-squared | 0.027 | 0.027 | 0.139 | 0.146 | 0.148 | 0.150 |

Notes: Standard errors are reported in parentheses, and are clustered at the village level. *, ** and *** indicate significance at the 10%, 5% and 1% level, respectively. See Section 2 for descriptions of the variables. All columns include controls for child age and sex. Columns 3-6 include village fixed effects, columns 4-6 include controls for household demographics and religion.
