# Supplementary material for: Is Exposure to Poultry Harmful to Child Nutrition? An Observational Analysis for Rural Ethiopia
Source: PLoS One. 2016 Aug 16;11(8):e0160590. doi: 10.1371/journal.pone.0160590 (PMC4986937; doi:10.1371/journal.pone.0160590)
Supplement: S5 Table — (DOCX) [file pone.0160590.s006.docx]

**S5 Table. Adjusted quantile regression models of child HAZ scores against binary indicators of "Owns poultry" and "Poultry in house"**

| **Estimator** | **OLS** | **Q25** | **Q50** | **Q75** | **Q25 vs Q75** |
| --- | --- | --- | --- | --- | --- |
|  | N=3,494 | N=3,494 | N=3,494 | N=3,494 | N=3,494 |
|  |  |  |  |  |  |
| Owns poultry (0/1) | 0.248*** | 0.149 | 0.209** | 0.279** | 0.130 |
|  | (0.082) | (0.121) | (0.093) | (0.127) | (0.141) |
| Poultry kept in house (0/1) | -0.252** | -0.241* | -0.291*** | -0.344** | -0.104 |
|  | (0.100) | (0.137) | (0.097) | (0.158) | (0.183) |
|  |  |  |  |  |  |
| All other controls included? | Yes | Yes | Yes | Yes | Yes |

Notes: *** p<0.01, ** p<0.05, * p<0.1. Bootstrapped (200 repetitions) standard errors in parentheses. Through necessity, these regressions control for region fixed effects rather than village fixed effects. The last column provides the results of inter-quantile regression that compares the coefficients between the 75th and 25th quantiles. In the unconditional HAZ distribution, the 25^th^ quantile corresponds to -3.07 HAZ score, 50^th^ quantile (median) to -1.94 HAZ score and 75^th^ quantile to -0.72 HAZ score.
